# Development of Antioxidant-Fortified Oleogel and Its Application as a Solid Fat Replacer to Muffin

**DOI:** 10.3390/foods10123059

**Published:** 2021-12-09

**Authors:** Sohui Jeong, Suyoung Lee, Imkyung Oh

**Affiliations:** 1 Department of Food Science & Technology, Sunchon National University, Sunchon 57922, Korea; thgml2502@naver.com; 2 Carbohydrate Bioproduct Research Center, Department of Food Science and Biotechnology, Sejong University, Seoul 05006, Korea; suyonglee@sejong.ac.kr

**Keywords:** fat replacement, oleogel, baking, micro-CT

## Abstract

Oleogelation has recently received a great deal of attention in the food industry as a novel alternative technology that physically converts liquid oil into semi-solid gel. Since the functional characteristics of oleogels are dependent on the gelators or bioactive compounds incorporated, this study was undertaken to evaluate the rheological properties and oxidative stability of candelilla wax oleogels fortified with glycerol monostearate (GMS) and β-carotene, and also to investigate their applications to muffin as a shortening replacer. The interaction between candelilla wax and GMS contributed to strengthening the oleogel structure. The oleogels with β-carotene showed the lowest peroxide values than the other samples. The muffins prepared with oleogels for shortening had greater specific gravity and harder texture, but there was no significant difference in the specific volume between the shortening and oleogel samples with GMS. In addition, muffins with β-carotene oleogels showed the highest oxidative stability. Therefore, this study indicated that the incorporation of β-carotene and GMS in oleogels positively affected the storage stability of muffin.

## 1. Introduction

Oleogel is usually generated by adding a structuring agent known as a gelator, which plays a role in creating a three dimensional network derived from the formation of Van der-Waals interaction and hydrogen bonds [1]. The solid-like properties of oleogels and their healthier fatty acid profile suggest that they could be used to replace the saturated fats (shortening, animal fat, margarine, etc.) [2]. Thus, there are previous reports that applied oleogels to food products for reducing the trans/saturated fat intake [3,4,5]. Nonetheless, the current oloegelation technology are still faced to many problems that have not been solved yet, such as shelf-life, consumer acceptance, desirable texture and mouthfeel.

In the recent studies on oleogelation, researcher started to explore the synergic effects of oleogel incorporating different oleogelators or bioactive compounds. Martins et al. [6] reported that a mixture of beeswax and β-carotene strengthened the structural conformation of oleogel, resulting in greater oxidative stability. Jana et al. [7] showed that the blending of two different waxes (beeswax, rice bran wax, or sunflower wax) in soybean oil changed the melting point and crystallization behavior. Interestingly, the mixing of different oleogelators can generate positive interactions and reduce the amount of gelator used to provide the desired texture as the solid form [8]. Since the presence of combined gelators lead to higher mechanical gel strength than single component oleogel [9]. Moreover, the interactions between oleogelators can change gel characteristics by governing the solubility balance. It is generally recognized that the solubility balance of hydrophobic and hydrophilic fraction of gelators, such as glycerol monostearate (GMS), sorbitan and monoacylglycerol, contributes to the structural arrangements and the formation of different three-dimensional crystal networks that stabilize the gel [10]. Out of these gelators, GMS is a hydrophobic, non-ionic, low molecular weight surfactant, and acts as a lipid structuring agent at relatively low concentrations [2,11]. Barroso et al. [12] examined the combination effects of GMS with berry wax or sunflower wax on oxidative stability and rheological properties of oleogels, and Choi et al. [13] reported that the oleogels prepared by the mixed blends of candelilla wax and GMS had lower melting points, which implied a eutectic behavior by showing increases in hardness and viscoelasticity. Since the interaction between wax-based gelators and GMS during oleogel preparation can cause the thermal, rheological, and morphological changes, it may be necessary to further enhance the functional properties of oleogels with GMS in food systems.

Bioactive compounds have also been used in preparing oleogels in order to improve the shelf life and nutritional value. Specifically, the combinations of antioxidants including vitamin C [14], proanthocyanidin [15], Co Q10 [16], and curcumin [17] in oleogels have been reported in various studies. As one of the most commonly available antioxidants, β-carotene is a lipid soluble compound that belongs to the well-known carotenoids with many health benefits [18]. Applications of β-carotene to oleogels have been reported by Martins et al. [6] and Chloe et al. [19]. However, there were many limitations on the practical use of β-carotene for industrial purposes because of low stability, bioaccessibility, and water solubility. For improving this demerit, the encapsulation of β-carotene by ethylcellulose and the preparation of protein based-emulsion oleogel with of β-carotene were conducted in several studies [19,20]. Moreover, the processing performance of oleogels with β-carotene in baking foods recently has been started to study. Chen et al. [20] indicated that zein-based emulgel with β-carotene was applied to sponge cake as a margarine alternatives. However, the study of synergic effect of wax-based oleogel when is formed with GMS and β-carotene has not been elucidated yet. The processing performance of oleogel combined with various oleogelators and β-carotene need to be investigated in order to expand the utility of fortified oleogel with β-carotene. Therefore, the primary research focus of this study was placed on the combination effects of two oloegelators (candelilla wax and GMS) and β-carotene on the rheological properties and oxidative stability of oleogels whose feasibility as shortening replacement in baking product were also investigated.

## 2. Materials and Methods

### 2.1. Preparation of Oleogels

Candelilla wax (Kahl GmbH and Co. KG, Trittau, Germany), β -carotene (Sigma-Aldrich, St. Louis, MO, USA), glycerol monostearate (GMS, Ilshinwells Co., Ltd., Seoul, Korea), and sunflower oil (Sajodaerim Co., Seoul, Korea) were purchased from a commercial market. Candelilla wax (10 g) was added to sunflower oil (90 g) and heated at 90 °C until the wax melted, as previously described by Kim et al. [21]. To prepare antioxidant-fortified oleogels, β-carotene (0.02%) was added to the oil suspension, and candelilla wax was replaced with GMS at the ratio of 1:3, which was determined from preliminary experiments [13]. The samples prepared were named as oleogel (O), oleogel with GMS (OG), oleogel with β-carotene (OB), and oleogel with GMS and β-carotene (OGB). The mixtures were cooled at room temperature for 30 min, placed in the freezer (−21 °C) for 1 h. Since the cooling rate is determined the crystal length and network pore area [22], the oleogel was cooled as possible as fast until the inner temperature reached about 5 °C. In addition, the oleogel was transferred to the refrigerator (5 °C) and stored further analysis.

### 2.2. Textural Measurement of Oleogels

The hardness of oleogel was measured using a texture analyzer (Sun Scientific Co., Ltd., Model CR_100, Setagayaku, Japan) with a 2 kg load cell at a speed of 60 mm/min using a cylindrical probe (5 mm diameter). Oleogel samples were subjected to compression at a strain of 50% after storage for 30 min at room temperature.

### 2.3. Rheological Measurement

The rheological properties of oleogels were measured by using a controlled stress rheometer (Discovery HR-2, TA Instrument, Nes Castle, DE, USA) equipped with a 40 mm parallel plate geometry at room temperature. The dynamic oscillatory storage (G′) and loss (G″) moduli of oleogels were measured in the frequency range 0.1–100 Hz at a strain 0.1%, which was within the linear viscoelastic limit. In addition, the viscoelastic changes of oleogels were measured by increasing temperature from 30 to 70 °C at a heating rate of 5 °C/min.

### 2.4. Oxidative Stability Measurement during Storage

The peroxide value was measured according to method of Cho et al. [23] and expressed in meq of peroxide/kg. Briefly, 1 g of each oleogel was dissolved with the solution of glacial acetic acid (15 mL) and chloroform (10 mL) (Daejung Chemicals and Metals Co., Ltd., Siheung, Korea), followed by the addition of saturated potassium iodine solution (1 mL). After left for 10 min in the dark, the mixture was blended with 30 mL of distilled water and then titrated against 0.01 N Na_2_S_2_O_3_ including 1% starch solution as an indicator. The end point was determined when the coloration by starch disappeared. The peroxide value of oleogel was analyzed every 7 days while stored at 60 °C for 28 days.

### 2.5. Preparation of Muffins

In order to assess the potential application of oleogels to baked goods, the shortening in muffins was replace with four different oleogels. The ingredients of muffin are as follows: 200 g wheat flour (CJ Co., Seoul, Korea), 130 g sugar (CJ Co., Seoul, Korea), 100 g water, 100 g whole egg, 100 g shortening (Lotte Co., Seoul, Korea), 16 g non-fat dry milk (Seoulmilk Co., Seoul, Korea), 4 g baking powder (Cheongeun F&B Co., Goyang, Korea), and 1 g salt (CJ Co, Seoul, Korea). The fat components (shortening and oleogels) were mixed with sugar for 2 min at speed 6 using a KitchenAid mixer (St Joseph, MI, USA). Whole eggs were added and blended for 3 min at speed 6. After the dry ingredients (wheat flour, non-fat dry milk, baking powder, and salt) were poured, they were mixed for 10 s at speed 2 and 50 s at speed 6, and then blended with water for 10 s at speed 2 and 50 s at speed 6. Each muffin batter (70 g) was contained into metal muffin pans and baked in a convectional baking oven (SMP-1010, Daeheung Softmeal Co. Ltd. Gwangju, Korea) at 185 °C for 28 min. The muffins were cooled for 1 h at ambient temperature.

### 2.6. Specific Gravity of Muffin Batter

The specific gravity of muffin batter was measured by dividing the weight of the batter in standard container by weight of water in the equal volume. Specific gravity was calculated following formula:

(1) $$\mathrm{Specific}\;\mathrm{gravity}=\frac{\mathrm{weight}\;\mathrm{of}\;\mathrm{muffin}\;\mathrm{batter}}{\mathrm{weight}\;\mathrm{of}\;\mathrm{an}\;\mathrm{equal}\;\mathrm{volume}\;\mathrm{of}\;\mathrm{water}}$$

### 2.7. Specific Volume of Muffin

The specific volumes of muffins were measured by using a laser topographic system (Volscan Profiler-VSP600, Stable Micro System Ltd., Surrey, UK) according to the method of AACC (10–16.01) [24]. Specific volume was determined at the ratio of the muffin volume and weight.

### 2.8. Tomographic Analysis of Muffin Crumb

The structural properties of muffins were non-destructively visualized by X-ray micro-computed tomography (micro-CT) (Skyscan 1174, Bruker, Kontich, Belgium). A cylindrically-shaped (30 mm diameter × 25 mm height) crumb sample was cut from the center of muffins. Micro-CT images were obtained on X-ray radiation generated from a 50 kV voltage and eletron current was set at 800 μA, and 1° angular interval during the 180° rotation. The 2D cross-sectional images (1024 × 1024 pixels) were analyzed by NRecon software (version 1.6.6, Bruker, Konitich, Belgium) and the total porosity was obtained from stacked 2D images using CTAn software (Skyscan series, Bruker, Billerica, MA, USA).

### 2.9. Textural Measurement

Texture properties of muffins were measured using a double compression cycle test that was carried out using a texture analyzer (Sun Scientific Co., Ltd., Model CR_100, Setagayaku, Japan) with 2 kg load cell. After removing the crusts, the muffin crumb was sliced into a cube shape (20 × 20 × 20 mm) and compressed at a crosshead speed of 60 mm/min to 60% of its original highest with a cylindrical probe of 50 mm diameter.

### 2.10. Color Measurement of Muffin

Muffin colors were determined by a colorimeter (Konica Minolta Sensing, Inc., Osaka, Japan) and expressed as L*, a*, b* values, where L* means lightness/darkness, a* means redness/ greenness, and b* means yellowness/blueness. Hue angle (H°) and chroma (C*) were calculated based on the following equations [25]:

(2) $$C^{*}=\sqrt{{\left(a^{*}\right)}^{2}+{\left(b^{*}\right)}^{2}\;}$$

(3) $$H{}^{\circ}=tan-1\frac{b^{*}}{a^{*}}\;$$

### 2.11. Oxidation Stability Measurement of Muffins

The storage stability of antioxidant-fortified oleogel in muffins was assessed under accelerated condition (60 °C, 14 days). After the muffins were ground using a blender, they were placed in a thimble filter and fat was extracted with ethyl ether for 7 h at 60 °C using the Soxhlet method. The oxidative stability of the muffins was described in terms of peroxide value.

### 2.12. Statistical Analysis

All experiments were performed in triplicate and the results were statistically analyzed with SPSS software (IBM SPSS Statistics 26, SPSS Inc., Chicago, IL, USA). Duncan’s multiple range tests were performed to determine the significant differences among samples at a confidence level of 95%

## 3. Results and Discussion

**Figure 1:**
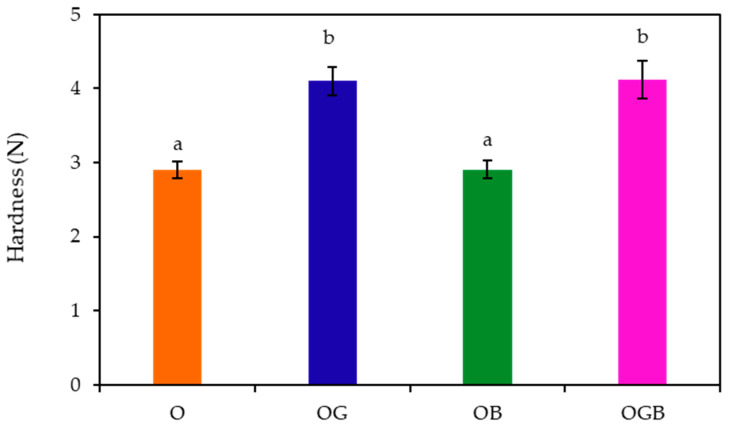
Hardness of oleogels with candelilla wax, GMS, and β-carotene (Means with different letters on the bars differ significantly at *p* < 0.05, O: oleogel, OG: oleogel + GMS, OB: oleogel + β-carotene, OGB: oleogel + GMS + β-carotene).

The hardness result of the oleogels was obtained from their maximum peak force values and presented in Figure 1. The highest values of hardness were observed in the OG and OGB samples and there were no significant differences between the two samples. Thus, the oleogel samples containing GMS exhibited significantly higher hardness (*p* < 0.05) than the other samples without GMS, indicating that the interaction between candelilla wax and GMS might contribute to reinforcing the oleogel structure. This tendency was consistent with the result of Choi et al. [13], who suggested that a combination of candelilla wax and GMS (3:1) increased the oleogel strength. Barroso et al. [12] also found that the oleogelator mixture of sunflower wax and GMS (1:1) affected the mechanical properties of gel. Thus, several recent studies have reported the combination effects of various oleogelators on the oleogel formation [12,26,27]. In the case of oleogels with β-carotene (OG and OGB), their hardness values were not significantly different from those without β-carotene (O and OG) (*p* < 0.05). Cui et al. [28] indicated that the ratio of β-carotene and GMS also influenced the development of oleogel. While the oleogel with 5% GMS could formed without the addition of β-carotene, there was no gel formation when β-carotene was added [28]. When β-carotene was mixed with other gelators, the ratio of blending, type of gelators and gelator concentrations could be considered with important factor because they can influence the oleogel mechanism. The textural properties of oleogel with can directly affect the physical and sensory property of foods, further studies on their correlations will be necessary.

**Figure 2:**
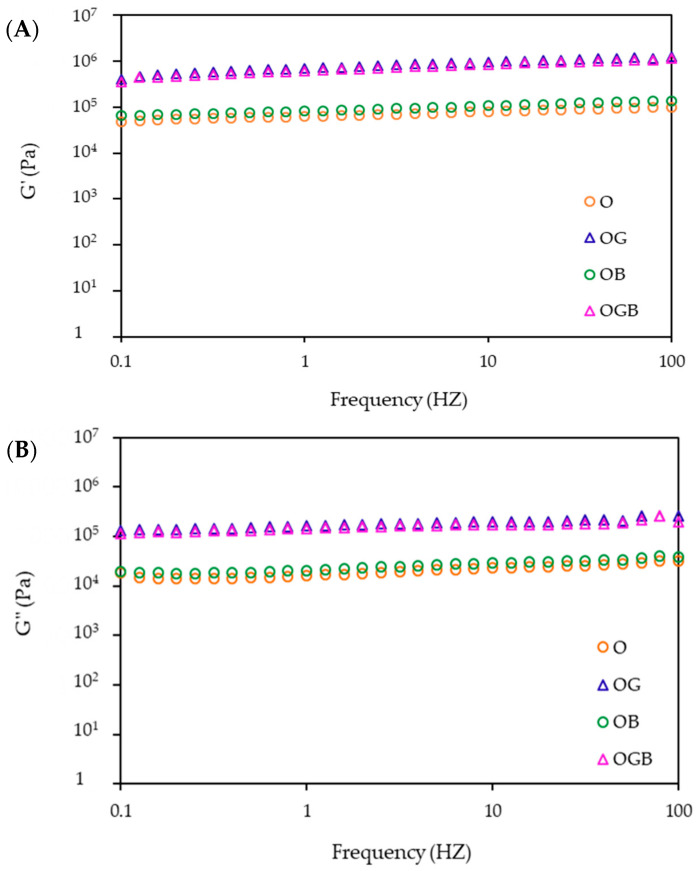
Dynamic viscoelastic properties of oleogels as a function of frequency: (**A**) storage moduli and (**B**) loss moduli (O: oleogel, OG: oleogel + GMS, OB: oleogel + β-carotene, OGB: oleogel + GMS + β-carotene).

A frequency sweep test was conducted to measure the storage modulus (G′, representing the elastic property) and the loss modulus (G″, depicting the viscous property) of the oleogels (Figure 2). All the samples showed dominate storage modulus over the loss modulus and both increased slightly over the frequency range, which represented a semi-solid behavior with good tolerance to deformation. When comparing the viscoelastic properties of O and OB samples with those of the OG and OGB samples, the oleogel samples with GMS were shown to have great G′ values (G′ > 10^5^) and this tendency was consistent with the hardness results. These results showed the synergistic effect of candelilla wax and GMS on the rheological characteristics of oleogels.

In contrast to Martins’ study [6] using bees wax (3%) and β-carotene, the effect of β-carotene on hardness and viscoelasticity was not observed in this study, which was used in the level of 10% candelilla wax concentration. This result could be related to the wax concentration. As the increase in wax concentration could be responsible for a different crystalline arrangement [6], β-carotene in surpassed wax concentration than gelling concentration seems not to affect the gel mechanisms.

**Figure 3:**
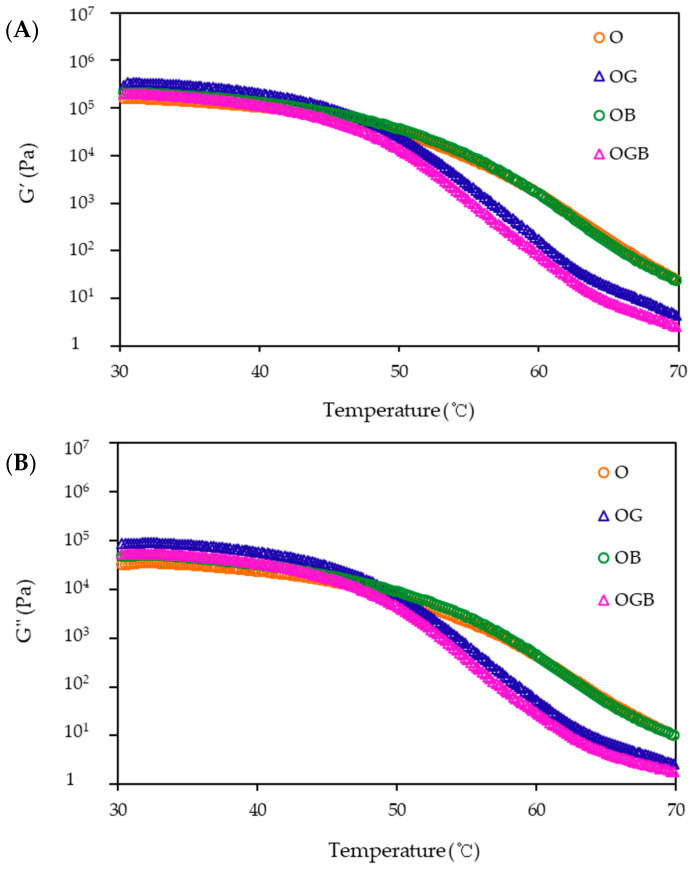
Viscoelastic changes of oleogels over temperature: (**A**) storage moduli and (**B**) loss moduli (O: oleogel, OG: oleogel + GMS, OB: oleogel + β-carotene, OGB: oleogel + GMS + β-carotene).

In order to investigate the effect of temperature on the mechanical behavior of oleogels, their dynamic viscoelastic properties were measured over temperature ranging from 30 to 70 °C (Figure 3). For all the oleogel samples, storage moduli (G′) were higher than loss moduli (G″), indicating a dominant elastic nature. As the temperature increased, the oleogel prepared with candelilla wax, GMS, and β-carotene (OGB) had the lowest values of viscoelastic parameters, followed by OG, OB, and O. At temperatures above 45 °C, GMS and candelilla wax-mixed oleogels had larger slopes, these rheological changes were related to the thermal characteristics. It was previously reported that the melting point of the oleogel prepared with GMS and candelilla wax was significantly lower (*p* < 0.05) than that of the candelilla wax oleogels [13]. The viscoelasticity change over temperature in this study could be explained based on the thermal characteristics such as melting point.

**Figure 4:**
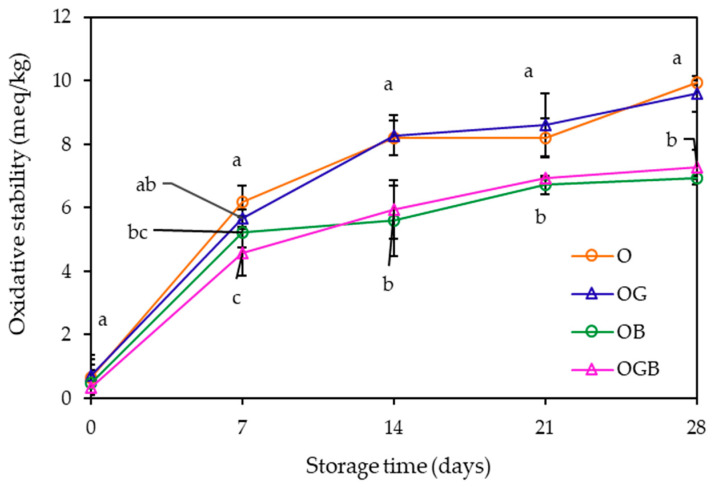
Oxidative stabilities of oleogels during storage (Means with different letters above the symbols differ significantly at *p* < 0.05, O: oleogel, OG: oleogel + GMS, OB: oleogel + β-carotene, OGB: oleogel + GMS + β-carotene).

The oxidative stability of oleogels was analyzed under the accelerated conditions by measuring their peroxide values, which indicates the amount of primary products formed during lipid oxidation. As can be seen in Figure 4, the peroxide values of the oleogels containing β-carotene (OB and OGB) were significantly lower (*p* < 0.05) than those of the others (O or OG), demonstrating that the addition of β-carotene could enhance the oxidative stability. Several studies concluded that the oleogelation can improve the oxidative stability compared to liquid oil [5,29,30]. This effect was attributed to the entrapment of oil in the gel structure formed, thereby retarding the oxidation by less oil exposure to air. In addition, Pan et al. [29] described that the denser network formation of oleogel showed the lower oxidation rate. However, as presented in this study, the addition of GMS produced oleogel with a harder texture, but there was no significant difference in the oxidative stability. On the other hand, the addition of β-carotene significantly improved (*p* < 0.05) the effect of the oxidative stability, probably due to the intrinsic antioxidant activity of β-carotene.

The specific gravities of muffin batters prepared with oleogels were compared with that of the shortening batter (Table 1). It is recognized that the specific gravity of batter decreases when more air cells are included [31]. Since air cells are entrapped in the fat phase of a batter system, the shortening in muffins plays an important role in increasing their volume and also generating an air cell structure [32]. As shown in Table 1, the replacement of shortening with oleogels tended to increase the specific gravity of batter, so the lowest specific gravity was observed for the sample with shortening. There were no significant differences in specific gravity among the oleogel samples. Therefore, it seemed that the replacement of shortening with oleogels negatively affected the air-entrapping capacity of the batters.

Specific volume is an important quality factor of bakery products to affect consumer preferences [33]. The effect of replacing shortening with oleogel on the muffin volume is shown in Table 1. The specific volumes of the muffins prepared with shortening and GMS oleogels were determined to be 1.71 and 1.62 mL/g. The muffin samples with oleogels without GMS showed lower specific volume that was measured to be 1.58 and 1.52 mL/g, respectively. The reduction in specific volume of muffins made with oleogel as a replacement for shortening may be related to the compact structure derived from the low air retention [34]. Even though the significant differences of the specific gravity were observed between shortening and OG (or OGB), it appeared that the use of oleogels with GMS did not have a negative effect on the specific volume of muffins.

The micro-CT images of muffin crumbs with different oleogels were analyzed by X-ray micro-CT (Table 1). In the micro-CT images, pore spaces were represented by black areas and solid fractions were represented by white areas [35,36]. As shortening was replaced with oleogels in the muffins, it seemed that larger and more air cells were observed, but no distinct differences were not observed among the muffin samples containing oleogels. Although muffins prepared with O or OB had significant difference in specific volume compared to control muffin (*p* < 0.05), their total porosity showed similarity with control muffin.

The visual appearance of muffin was presented Table 2. Muffins with β-carotene containing oleogels displayed a more yellow color than the others, which could be due to the β-carotene. The shortening muffin displayed a uniform distribution of small pores, while the oleogels cakes displayed a non-uniform distribution of relatively large pores in a dense network of muffin matrix. After 14 days storage, the quality losses such as color changes and mold were not observed in muffin samples.

The textural properties for the muffins prepared with shortening and oleogels were compared. As presented in Table 3, the muffin prepared with shortening exhibited the lowest hardness, compared to the muffins with oleogels probably due to its structural features as shown in Table 1. Furthermore, the muffins with the GMS oleogels were softer than those without GMS. Preceding studies [37,38] reported that the muffins where shortening was replaced oleogel, had a harder texture. The color parameters of the oleogel muffins are presented in Table 4. Redness (a*) and yellowness (b*) values of the muffin samples (crust) were significantly (*p* < 0.05) increased by replacement of shortening with oleogels containing β-carotene. As also can be seen in Table 4, Hue angle (H°) and chroma (C*) that are a measure of color nuance and purity of color, respectively, tended to significantly increase in the muffins containing β-carotene (*p* < 0.05). The lowest lightness (L*) and the highest and redness (a*) and yellowness (b*) were obtained at the OB sample. This phenomenon indicated that the presence of β-carotene in the oleogel formulation affected the color of the muffins.

**Figure 5:**
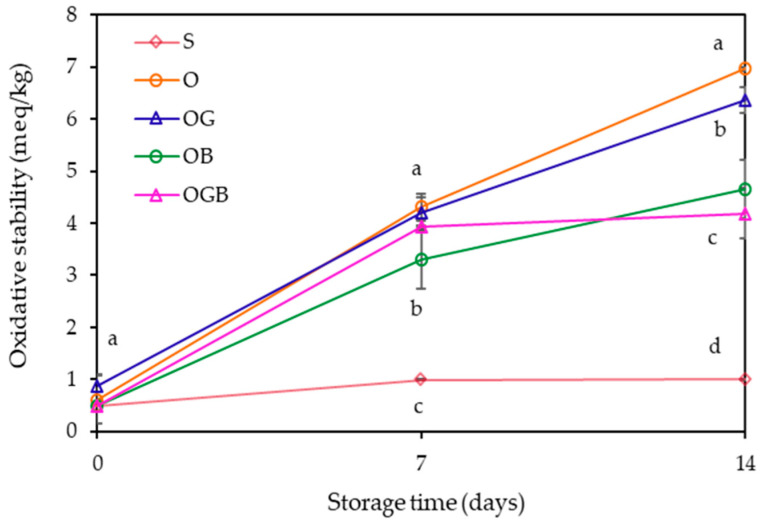
Effects of shortening replacement with oleogles on the oxidative stability of muffins (Means with different letters above the symbols differ significantly at *p* < 0.05, O: oleogel, OG: oleogel + GMS, OB: oleogel + β-carotene, OGB: oleogel + GMS + β-carotene).

One of the main problems of the food products with a high level of unsaturated oil is the quality loss derived from oil oxidation during storage. Hence, in the current study, the peroxide values of muffins were monitored for 14 days under accelerated conditions (Figure 5). The lowest peroxide value was observed in the muffins prepared with shortening. Among the oleogel samples, the OGB muffin sample showed the lowest peroxide value, followed by OB, OG, and O muffins. The peroxide values of muffin with β-carotene (OG and OGB muffins) were significantly decreased. After 14 days, the peroxide value of the OG muffin became lower than that of the O muffin, although there was no significant difference in the oxidative stability of oleogel. As presented in this study, the oleogel fortified with β-carotene seemed to be more effective in increasing the oxidative stability of muffins than the GMS oleogel.

## 4. Conclusions

The results obtained from this study showed that the use of GMS with candelilla wax increased the rheological properties of oleogels such as gel strength and viscoelasticity. The oleogels prepared with GMS and β-carotene resulted in the lowest peroxide value and had the highest oxidative stability. When the oleogel with GMS and β-carotene (OGB) was applied for muffin, there was no significant difference in specific volume and total porosity compared to shortening muffin. OGB muffin showed the lowest hardness and oxidative stability among the other oleogels. Thus, this study indicated that antioxidant-fortified oleogels could be used as replacement for shortening to develop bakery products with lower saturated and trans-fatty acid levels.

## Figures and Tables

**Table 1:** Effect of shortening replacement with oleogles on the specific gravity of muffin batter and specific volume, cross-sectional micro-CT images, and total porosity of muffins.

	S	O	OG	OB	OGB
Specific gravity of batter	0.97 ± 0.00 b	1.13 ± 0.00 a	1.13 ± 0.01 a	1.13 ± 0.01 a	1.12 ± 0.01 a
Specific volume of muffin (mL g^−1^)	1.71 ± 0.02 a	1.58 ± 0.11 bc	1.62 ± 0.01 abc	1.52 ± 0.06 c	1.65 ± 0.02 ab
Cross-sectional micro-CT image of muffin	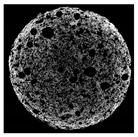	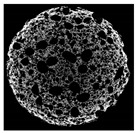	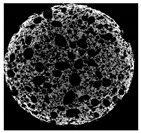	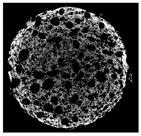	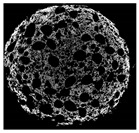
Total porosity (%)	63.91 ± 2.85 a	62.89 ± 1.90 a	62.79 ± 1.28 a	62.45 ± 1.43 a	62.16 ± 1.80 a

Samples were named oleogel (O), oleogel with GMS (OG), oleogel with β-carotene (OB), and oleogel with GMS and β-carotene (OGB). Values are means ± standard deviation (*n* = 3). Means with different small letters in the same column differ significantly by Duncan test (*p* < 0.05).

**Table 2:** Effect of shortening replacement with oleogels on the visual appearances of muffins.

Visual Appearance of Muffin	S ^1^	O	OG	OB	OGB
0 day (before storage)	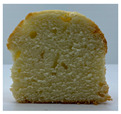	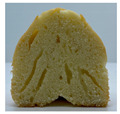	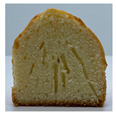	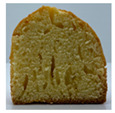	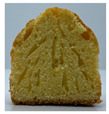
14 day (after storage)	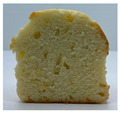	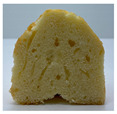	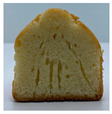	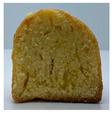	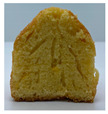

**^1^** Samples were named oleogel (O), oleogel with GMS (OG), oleogel with β-carotene (OB), and oleogel with GMS and β-carotene (OGB).

**Table 3:** Texture analysis results of muffins containing fortified-oleogel as a shortening replacer.

Texture	S ^1^	O	OG	OB	OGB
Hardness (N)	2.34 ± 0.48 d ^2,3^	10.16 ± 0.38 b	7.92 ± 0.87 c	11.49 ± 1.01 a	7.89 ± 0.71 c
Springiness (mm)	0.86 ± 0.01 a	0.85 ± 0.03 a	0.78 ± 0.01 b	0.86 ± 0.01 a	0.81 ± 0.02 b
Cohesiveness	0.78 ± 0.07 a	0.65 ± 0.02 b	0.53 ± 0.02 c	0.64 ± 0.01 b	0.58 ± 0.01 c
Chewiness (J)	1.84 ± 0.29 e	6.50 ± 0.29 b	3.69 ± 0.52 d	7.87 ± 0.52 a	4.40 ± 0.34 c

**^1^** Samples were named oleogel (O), oleogel with GMS (OG), oleogel with β-carotene (OB), and oleogel with GMS and β-carotene (OGB). ^2^ Values are means ± standard deviation (*n* = 3). ^3^ Means with different small letters in the same column differ significantly by Duncan test (*p* < 0.05).

**Table 4:** Color of muffins containing fortified-oleogel as a shortening replacer.

Color	S ^1^	O	OG	OB	OGB
L*	61.63 ± 0.49 d ^2,3^	63.22 ± 0.37 b	63.84 ± 0.46 a	60.97 ± 0.27 e	62.54 ± 0.31 c
a*	9.79 ± 0.30 b	9.31 ± 0.22 c	9.51 ± 0.28 bc	11.19 ± 0.26 a	10.95 ± 0.28 a
b*	28.01 ± 0.37 c	27.85 ± 0.42 c	26.86 ± 0.31 d	50.06 ± 0.47 a	47.86 ± 0.14 b
C*	29.67 ± 0.44 c	29.60 ± 0.43 c	28.50 ± 0.32 d	51.30 ± 0.52 a	49.09 ± 0.09 b
H°	70.75 ± 0.33 c	71.51 ± 0.39 b	70.51 ± 0.53 c	77.40 ± 0.18 a	77.11 ± 0.35 a

**^1^** Samples were named oleogel (O), oleogel with GMS (OG), oleogel with β-carotene (OB), and oleogel with GMS and β-carotene (OGB). ^2^ Values are means ± standard deviation (*n* = 3). ^3^ Means with different small letters in the same column differ significantly by Duncan test (*p* < 0.05).

## Data Availability

The datasets generated for this study are available on request to the corresponding author.
